# Differential P3 reactivity to pornographic versus natural rewards as a neural marker of reward imbalance in problematic pornography use in young heterosexual men

**DOI:** 10.1016/j.ijchp.2026.100710

**Published:** 2026-07-24

**Authors:** Yuanyuan Liu, Siying Zhong, Jianfeng Wang

**Affiliations:** School of Psychology, Chengdu Medical College, Chengdu, China

**Keywords:** Problematic pornography use, Reward dysregulation, Cue reactivity, Natural rewards, P3

## Abstract

Problematic pornography use (PPU) has been increasingly recognized as a behavioral addiction involving aberrant reward processing. However, few studies have examined reward imbalance—the co-occurrence of heightened reactivity to pornographic cues and reduced responsiveness to natural rewards—as a putative mechanism underlying PPU. The present study investigated whether the differential P3 component (ΔP3; pornographic minus natural reward P3) could serve as a neural index of individual differences in PPU risk in a sample of young heterosexual men. A total of 139 participants (aged 18–25 years, *M* = 20.54, *SD* = 1.53) completed an ERP oddball task and questionnaires assessing both pornography use and PPU symptoms. Results showed that ΔP3 was positively associated with both pornography use and PPU severity and demonstrated moderate discriminative performance in distinguishing high- versus low-risk participants (AUC = 0.71). In contrast, pornographic reward P3 (PR-P3) and natural reward P3 (NR-P3) alone showed weak and non-significant associations with outcome measures. These findings suggest that reward dysregulation indexed by ΔP3 may reflect a neural signature of altered reward processing in PPU, providing preliminary support for reward imbalance models of addictive behaviors. More broadly, the results highlight the potential value of comparative neural indices over single-cue reactivity measures in characterizing individual differences in PPU.

## Introduction

The rapid development of internet technology has dramatically changed the landscape of sexual stimulation, making pornography more accessible, anonymous, and affordable than ever before. While the majority of individuals consume pornography occasionally for entertainment without experiencing functional impairment (Campbell & Kohut, 2017; Malki et al., 2021), a notable minority develop persistent and uncontrollable problematic pornography use (PPU). PPU is characterized by compulsive consumption of pornography, such as excessive preoccupation with pornography, repeated unsuccessful attempts to reduce use, continued use despite negative consequences, and reduced engagement in other rewarding activities (Kafka, 2010; Wéry & Billieux, 2017). Recent epidemiological studies indicate that the prevalence of PPU ranges from 3% to 10% among men and 1% to 7% among women (Bőthe et al., 2020; Bőthe et al., 2024; Grubbs et al., 2019). PPU, which is a subtype of compulsive sexual behavior disorder (CSBD), has been officially recognized in the ICD-11 as an impulse control disorder, highlighting its clinical and public health significance (World Health Organization, 2020; Antons & Brand, 2021). However, growing empirical evidence supports conceptualizing CSBD and PPU within an addiction framework, rather than viewing them solely from an impulse-control perspective (Brand et al., 2025; Klein et al., 2022; Kowalewska et al., 2018; Stark et al., 2018). Despite increasing research, the core neurobiological mechanisms underlying PPU remain insufficiently understood, hindering the development of targeted interventions.

A large body of neurobiological models supports the view that addiction is fundamentally characterized by imbalanced reward processing (Berridge & Robinson, 2016; Goldstein & Volkow, 2011; Koob & Volkow, 2016). This central framework suggests that addictive behaviors arise from two complementary alterations in the brain’s reward system: heightened salience of addiction-related stimuli and blunted responsiveness to natural rewards. This dual-pathway model is supported by several influential theories of addiction. The incentive sensitization theory proposes that repeated exposure to addictive stimuli progressively enhances the salience of addiction-related cues, transforming them into powerful motivational magnets that elicit intense craving and compulsive approach behaviors (Robinson & Berridge, 1993). The impaired response inhibition and salience attribution (IRISA) model extends this framework by emphasizing that addiction involves both hyper-reactivity to addictive cues and hypo-reactivity to natural rewards, which together disrupt inhibitory control and lead to maladaptive decision-making (Goldstein & Volkow, 2011). Similarly, the reward deficiency hypothesis suggests that inherently reduced sensitivity to natural rewards predisposes individuals to seek highly stimulating artificial rewards, such as drugs or compulsive behaviors, to compensate for dysfunctional reward circuits (Blum et al., 2012).

While incentive-sensitization models emphasize the heightened salience of addiction-related rewards and reward-deficit models focus on the reduced responsiveness to natural rewards, neither framework directly examines whether the differential valuation of these two reward types serves as a distinct risk marker for addiction. Instead, a set of complementary perspectives, including behavioral and neuroeconomic models of choice (Field et al., 2020; Monterosso et al., 2012) and computational neuroscience accounts of relative reward coding (Vlaev et al., 2011), suggests that the relative difference between addiction-related and natural rewards is critical to addiction etiology. Behavioral economic and value-based decision-making frameworks emphasize that reward-seeking behavior is guided by the relative value of options, rather than absolute reward magnitude (Field et al., 2020). These models propose that the brain encodes a subjective value signal for each option, with addiction involving a biased valuation toward addiction-related rewards (Bickel et al., 2011; Monterosso et al., 2012). Computational neuroscience models extend this by formalizing how relative reward differences are dynamically updated via reinforcement learning. Chronic exposure to addictive stimuli progressively widens the gap between addiction-related and natural reward values (Vlaev et al., 2011). Together, these frameworks suggest that the relative imbalance between addiction-related and natural rewards, rather than either system alone, confers vulnerability to addiction.

Empirical evidence from diverse addictive disorders consistently supports the reward imbalance framework. In substance use disorders such as cocaine, alcohol, and nicotine addiction, individuals show enhanced neural responses to drug-related cues and diminished reactivity to natural rewards, such as monetary, social, or emotional stimuli (Dunning et al., 2011; Goldstein et al., 2008; Versace et al., 2012). Importantly, this pattern of imbalance is not unique to substance addiction but extends to behavioral addictions, such as gaming and gambling disorders (Zhou et al., 2025; Zhou et al., 2021; Sescousse et al., 2013). For instance, individuals with gambling disorder show heightened sensitivity to monetary rewards and reduced responsiveness to other natural rewards, reflecting a similar pattern of prioritizing addiction-relevant over alternative reinforcers (Sescousse et al., 2013). These findings suggest that reward imbalance may represent a transdiagnostic marker underlying the development and maintenance of addictive behaviors.

Existing studies in PPU research have largely focused on heightened neural responses to pornographic cues (Lichte et al., 2025). Evidence from fMRI and event-related potential (ERP) studies shows that individuals with PPU exhibit increased attentional bias, stronger ventral striatum activation, and larger ERP amplitudes in response to sexual images (Draps et al., 2024; Gola et al., 2017; Voon et al., 2014; Wang et al., 2021), and these hyper-reactive responses are closely associated with the severity of PPU symptoms (Brand et al., 2016). However, another core component of reward imbalance—blunted processing of non-pornographic natural rewards—has been largely overlooked. To date, studies examining non-pornographic reward function in PPU remain limited and inconsistent. Previous work has noted that individuals with greater cue reactivity to pornographic stimuli often report a reduced desire for real-world partnered sexual activity (Steele et al., 2013), indicating a clear preference for artificial sexual cues over natural sexual rewards. In addition, emerging evidence suggests that individuals with PPU show reduced sensitivity to monetary rewards and pleasant non-sexual stimuli compared to healthy controls (Wang & Li, 2023; Wang et al., 2024). However, some fMRI studies reported no significant group differences in neural activation during monetary reward anticipation (Gola et al., 2017; Golec et al., 2021). Most importantly, no prior research has directly investigated the central feature of reward imbalance by comparing neural responses to pornographic and non-pornographic natural rewards in PPU.

The P3 component represents an ideal electrophysiological index for investigating reward imbalance in PPU. As a well-characterized ERP component, P3 peaks approximately 300–600 ms post-stimulus, is predominantly distributed over the parietal cortex, and reflects the processing of stimulus salience, motivational significance, and reward valuation (Polich, 2007; Hajcak & Foti, 2020). Extensive research has confirmed that P3 amplitude increases with the motivational salience and value of rewards, making it a reliable and sensitive marker for quantifying reward processing (Yeung & Sanfey, 2004; Pfabigan et al., 2014). In addiction research, P3 has been widely used to assess both hyper-responsiveness to addiction cues and hypo-responsiveness to natural rewards (Kohen et al., 2024; Goldstein et al., 2008; Parvaz et al., 2012; Wang et al., 2024; Webber et al., 2022). The differential P3 response between these two types of rewards serves as a robust neural marker of reward dysregulation (Martins et al., 2022).

Based on the above theoretical framework and empirical gaps, this study aims to examine reward imbalance in individuals at risk for PPU by comparing neural responses (P3 amplitude) to pornographic rewards and non-pornographic natural rewards. We hypothesize the following:

H1: Pornographic Reward-P3 (PR-P3) amplitude would be positively associated with pornography use and PPU symptom severity.

H2: Natural Reward-P3 (NR-P3) amplitude would be negatively associated with pornography use and PPU symptom severity.

H3: The ΔP3 (the difference between PR-P3 and NR-P3) would show stronger associations with pornography use and PPU severity than either single index, and would better distinguish individuals with high versus low PPU risk.

## Methods

### Participants

A priori power analysis was conducted using G*Power 3.1.9.2 (Faul et al., 2009) to determine the minimum sample size for linear regression. Setting the effect size at f² = 0.0625 (corresponding to *r* = 0.25)—an effect magnitude consistent with previous ERP research (Wang & Li, 2023; Wang et al., 2024)—the analysis yielded a required sample size of 128 participants, with an alpha level of 0.05 and statistical power of 0.80. A total of 153 male undergraduate students were initially enrolled in the present ERP study. Eleven participants were excluded due to excessive electrophysiological artifacts, and three more were excluded because of equipment malfunction during data acquisition. The demographic and clinical characteristics of the final sample of 139 individuals are summarized in Table 1. This study was approved by the local Ethics Committee. Written informed consent was obtained from all participants before participation.

**Table 1:** Participant demographics and clinical measures (Mean ± SD).

Variables	**Overall** **(n = 139)**	**High risk** **(n = 28)**	**Low risk** **(n = 111)**	***t/χ²***	***p***	***d/φ***
Age (years)	20.54 (1.53)	20.57 (1.73)	20.53 (1.48)	0.12	0.902	0.03
PPU symptoms	49.99 (21.40)	83.79 (8.92)	41.46 (13.80)	15.41	**<0.001**	**3.26**
Pornography use	81.92 (72.06)	145.36 (100.52)	65.92 (52.46)	5.80	**<0.001**	**1.23**
PR-P3	13.11 (5.69)	14.89 (4.63)	12.66 (5.87)	1.87	0.063	0.40
NR-P3	5.37 (4.75)	4.54 (5.17)	5.58 (4.64)	–1.03	0.305	0.22
ΔP3	7.74 (4.31)	10.35 (3.47)	7.08 (4.26)	3.76	**<0.001**	**0.79**
Anxiety (BAI)	6.04 (4.81)	7.93 (4.98)	5.56 (4.67)	2.37	**0.019**	**0.50**
Depression (BDI-II)	7.01 (5.59)	8.39 (5.13)	6.67 (5.67)	1.47	0.145	0.31
Alcohol use (at least once per month)	51/0.37 a	12/0.43 a	39/0.35 a	0.58	0.448	0.06
AUDIT	3.65 (1.65)	3.50 (2.20) b	3.69 (1.47) c	–0.35	0.727	0.12
Cigarette use (at least once per month)	36/0.26 a	5/0.18 a	31/0.28 a	1.27	0.260	0.10
FTND	2.72 (1.34)	2.60 (1.82) d	2.74 (1.29) e	–0.22	0.830	0.10
Gaming disorder (IGDS)	11.27 (6.57)	12.54 (6.05)	10.95 (6.68)	1.15	0.254	0.24
Gambling disorder (PGSI)	0.55 (1.18)	0.79 (1.07)	0.49 (1.20)	1.21	0.230	0.26

*Note*. AUDIT, Alcohol Use Disorder Identification Test; BAI, Beck Anxiety Inventory; BDI-II, Beck Depression Inventory-II; FTND, Fagerstrom Test for Nicotine Dependence; IGDS, Internet Gaming Disorder Scale; PGSI, Problem Gambling Severity Index; PPU, Problematic Pornography Use; PPCS, Problematic Pornography Consumption Scale; Pornography use = weekly viewing frequency × average duration per session; PPU symptoms = score on the Problematic Pornography Consumption Scale (PPCS); PR-P3 = Pornographic Reward-P3; NR-P3 = Natural Reward-P3; ΔP3 = difference between PR-P3 and NR-P3.

a Number of participants (percentage)

b n=12

c n=39

d n=5

e n=31 Bold font indicates statistical significance

### Recruitment

Participants were recruited via campus promotional advertisements, peer referrals, and online recruitment postings. The sample was limited to male individuals, consistent with evidence showing a markedly higher prevalence of PPU among males (Bőthe et al., 2024; Grubbs et al., 2019). Eligibility screening was conducted through a comprehensive online survey battery. Inclusion criteria required participants to be aged ≥18 years and to have reported at least one instance of pornography use within the past 6 months. Multiple exclusion criteria were applied to minimize confounding variables. These criteria included: hazardous alcohol consumption (AUDIT ≥8; Babor et al., 2001), nicotine dependence (FTND ≥6; Fagerström, 1978), internet gaming disorder (IGDS ≥36; Pontes & Griffiths, 2015), gambling disorder (PGSI ≥8; Ferris & Wynne, 2001), severe depressive symptoms (BDI-II ≥29; Beck et al., 1996), severe anxiety symptoms (BAI ≥26; Beck et al., 1988), current or prior illicit drug use, and a documented history of neurological or psychiatric disorders. Non-heterosexual individuals were excluded because the erotic stimulus set used in this paradigm was validated solely for heterosexual males, lacking ecological validity for individuals with other sexual orientations. Eligible participants attended a laboratory session to complete the ERP task protocol.

## Questionnaire assessments

### Pornography use

The weekly duration of pornography use (in minutes) was assessed using two self-report items from Antons & Brand (2018). Participants reported their average weekly frequency of pornography consumption and the typical duration of each viewing session. Total weekly pornography use was calculated by multiplying the weekly viewing frequency by the average duration per session.

### Symptom severity of problematic pornography use

PPU was assessed using the Problematic Pornography Consumption Scale (PPCS; Bőthe et al., 2018), an 18-item instrument capturing six core components of problematic use: salience, mood modification, conflict, tolerance, relapse, and withdrawal. Items are rated on a seven-point Likert scale ranging from 1 (never) to 7 (always). Higher total scores reflect greater severity of PPU, with scores of 76 or above indicating risk for PPU. In the present sample, the PPCS showed excellent internal reliability (Cronbach’s α = 0.96).

### Screening measures

To determine eligibility and apply exclusion criteria, participants completed several standardized self-report instruments: Beck Depression Inventory–II (BDI-II; Beck et al., 1996), Beck Anxiety Inventory (BAI; Beck et al., 1988), Fagerström Test for Nicotine Dependence (FTND; Fagerström, 1978), Alcohol Use Disorders Identification Test (AUDIT; Babor et al., 2001), Internet Gaming Disorder Scale (IGDS; Pontes & Griffiths, 2015), and the Problem Gambling Severity Index (PGSI; Ferris & Wynne, 2001). Detailed descriptions of these measures are provided in the Supplementary Material.

### Picture-viewing oddball task

**Fig. 1:**
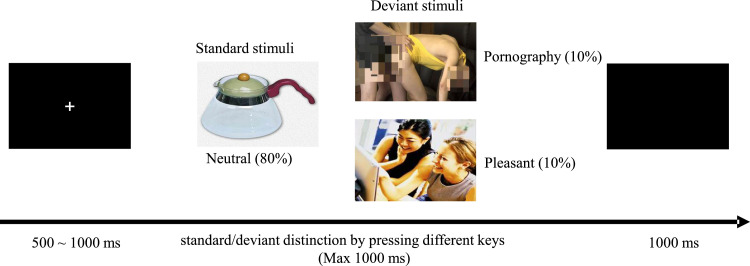
Schematic of the Picture-Viewing Oddball Task. Standard stimuli (80%) consist of neutral images, while deviant stimuli (10% each) include pornography and pleasant images. Participants distinguish between neutral and non-neutral stimuli by pressing different keys.

A modified visual oddball paradigm was adopted in the present ERP study (Fig. 1). The paradigm comprised five experimental blocks, with 100 trials per block. Neutral everyday objects (e.g., furniture, stationery) served as frequent standard stimuli (80%), and two categories of infrequent deviant stimuli, pleasant (e.g., landscapes, smiling faces) and pornographic scenes (sexually explicit heterosexual content), were presented at 10% each. Each trial commenced with a variable fixation cross displayed for 500–1000 ms. After the fixation period, a stimulus image was presented for 1000 ms. Participants were instructed to make speeded manual responses, pressing the F key for neutral standard stimuli and the J key for non-neutral deviant stimuli. The intertrial interval was 1000 ms. Stimuli were presented in a fully randomized order, and key-response mappings were counterbalanced among participants. Prior to the formal experiment, 10 practice trials were completed to familiarize participants with the task procedures.

The stimulus pool consisted of 25 pornographic images, 25 pleasant images, and 50 neutral images. Each pornographic and pleasant image was presented twice, while each neutral image was presented eight times throughout the task. Pornographic stimuli were obtained from publicly accessible adult websites and have been utilized in previous empirical studies (Wang et al., 2021; Wang et al., 2024). Neutral and positive affective pictures were selected from the Chinese Affective Picture System (Bai et al., 2005). All images were evaluated in a pilot study using 9-point Likert scales for valence (1 = very unpleasant, 9 = very pleasant) and arousal (1 = not arousing, 9 = highly arousing). Detailed stimulus properties are provided in the Supplementary Materials.

### EEG recording and ERP data processing

Electroencephalographic (EEG) activity was recorded via a 64-channel Ag/AgCl electrode cap using a Brain Products recording system (Brain Products GmbH, Germany). The EEG signal was digitized at a sampling rate of 500 Hz, with an online bandpass filter set between 0.05 and 100 Hz. The FCz electrode was used as the online reference channel. Vertical electrooculographic (VEOG) activity was also recorded from an electrode positioned beneath the right eye. Throughout data acquisition, all electrode impedances were kept below 5 kΩ.

Offline EEG data preprocessing was performed in BrainVision Analyzer 2.1 software. Recordings were re-referenced to the average activity of the bilateral mastoid electrodes and filtered with a 0.1–30 Hz bandpass filter. Independent component analysis (ICA) was applied to correct ocular artifacts. Eye blink and eye movement components were identified and discarded based on their typical scalp distribution topographies. Artifactual epochs were rejected according to strict artifact rejection criteria, including voltage shifts exceeding 50 μV/ms, amplitude fluctuations greater than 100 μV within 200 ms, and absolute signal amplitudes exceeding ±100 μV. The average valid trial numbers for pornographic and pleasant conditions were 39.15 (*SD* = 9.72) and 38.44 (*SD* = 10.50), respectively.

ERPs were averaged exclusively from correct response trials across each stimulus condition. The analysis epoch spanned 1000 ms, with a 200 ms pre-stimulus baseline interval prior to stimulus onset. Based on visual inspection of the grand-averaged ERP waveforms and prior evidence from analogous picture-viewing paradigms (e.g., Martins et al., 2022), the P3 component exhibited maximal amplitudes at parietal electrode sites within a 350–600 ms post-stimulus window. Accordingly, P3 amplitudes were quantified as the mean voltage across the 350–600 ms interval, averaged over P3, Pz, and P4 parietal electrodes. Split-half reliability with Spearman–Brown correction indicated satisfactory reliability for both PR-P3 and NR-P3 (*r*s = .84 and .81, respectively). ΔP3 showed acceptable reliability (*r* = .66), consistent with prior reports for analogous neural difference indices of reward processing (Joyner et al., 2019; Luking et al., 2017).

### Statistical analysis

To examine associations between PR-P3, NR-P3, and their difference score (ΔP3) with pornography use and PPU symptoms, preliminary analyses computed zero-order correlations among all variables. Hierarchical linear regression analyses were then conducted to test predictive effects. Anxiety and depression were entered in Step 1. Pornography use was additionally included in Step 2 when PPU symptoms served as the dependent variable. In Step 3, PR-P3, NR-P3, and ΔP3 were entered in separate models. Incremental predictive validity was evaluated using changes in explained variance (ΔR²) and associated *F*-change tests.

Receiver operating characteristic (ROC) analyses were performed to evaluate the classification performance of PR-P3, NR-P3, ΔP3, and pornography use in distinguishing high- versus low-risk PPU based on PPCS scores. To address class imbalance, robustness of AUC estimates was assessed using non-parametric outcome-stratified bootstrapping (2,000 resamples preserving the original group ratio), providing 95% confidence intervals for AUC, sensitivity, and specificity. Classification stability was further evaluated using repeated stratified five-fold cross-validation (200 repetitions), yielding out-of-sample AUC estimates and stable performance across resamples. As a complementary imbalance-sensitive metric, precision–recall AUC (average precision) was computed for all predictors. Group differences in AUC were tested using DeLong’s test for correlated ROC curves, with confidence intervals for AUC differences derived from bootstrap procedures and p-values adjusted using Holm correction for multiple comparisons. Sensitivity, specificity, positive predictive value (PPV), and negative predictive value (NPV) were computed across multiple cut-off thresholds (+0.5, +1.0, +1.5, and +2.0 SD). Analyses were conducted in SPSS 26.0 and R 4.5.1 using the pROC package.

## Results

### Participant characteristics and P3 component analyses

Participant characteristics are summarized in Table 1. Of the 139 participants, 28 (20.1%) met criteria for high-risk PPU based on PPCS scores (≥76), with the remaining 111 classified as low-risk. The high-risk group reported significantly higher anxiety levels (Cohen’s d = 0.50), whereas no group differences were observed for age, depression, or other behavioral addictions (all *ps* > .05).

**Fig. 2:**
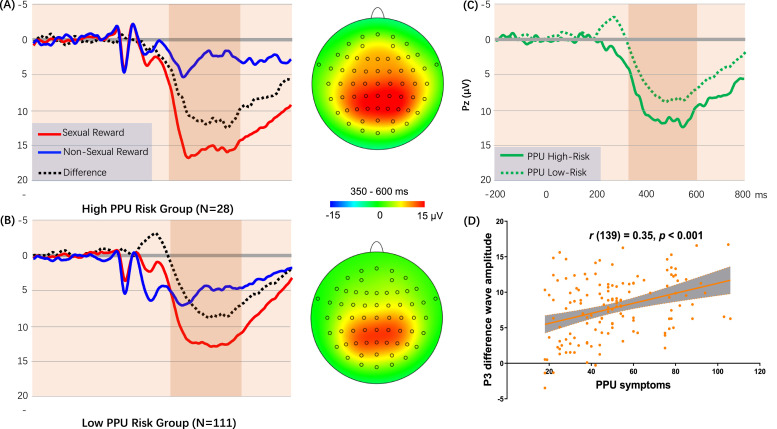
ERP waveforms and topographical distributions for P3 responses. (A) High-risk PPU group’s ERP response to sexual and pleasant rewards, showing the differential P3 (ΔP3) between the two stimuli types. (B) Low-risk PPU group’s ERP response to sexual and pleasant rewards, with ΔP3. (C) Comparison of ΔP3 responses between high-risk and low-risk PPU groups. (D) Scatter plot showing the positive correlation between ΔP3 amplitude and PPU symptom severity.

ERP analyses confirmed the expected modulation of the P3 component, which was maximal over parietal sites between 350–600 ms. Across conditions, emotional stimuli elicited larger P3 amplitudes than neutral stimuli, and pornographic images elicited greater P3 responses than pleasant images (pornographic: *M* = 13.11 μV, *SD* = 5.69; pleasant: *M* = 5.37 μV, *SD* = 4.75; neutral: *M* = 3.89 μV, *SD* = 4.28). Group comparisons revealed a marginal increase in PR-P3 in the high-risk group (Cohen’s d = 0.40), whereas NR-P3 did not differ significantly between groups (Cohen’s d = 0.22). In contrast, the differential index (ΔP3) showed a robust group effect, with higher values in the high-risk group (Cohen’s d = 0.79; Fig. 2).

### Predicting pornography use and PPU symptoms

Correlations between P3 measures and pornography-related outcomes are reported in Table 2. As expected, PR-P3 and NR-P3 were significantly positively correlated (*r* = .67, *p* < .001). PR-P3 was not correlated with pornography use, but showed a weak positive correlation with PPU symptoms (*r* = .20, *p* = .02); in contrast, NR-P3 showed no significant correlations with any pornography-related outcomes. Importantly, however, the difference score (ΔP3) was significantly positively correlated with both pornography use and PPU symptoms (*r* = .22, *p* = .009; *r* = .35, *p* < .001, respectively) (see Fig. 2). Additionally, anxiety and depression scores were weakly but significantly positively correlated with pornography-related outcomes (see Table S1).

**Table 2:** Intercorrelations between PR-P3, NR-P3, ΔP3, and pornography-related outcomes.

Variables	Pornography use	PPU symptoms	PR-P3	NR-P3	ΔP3
Pornography use	–				
PPU symptoms	.57⁎⁎⁎	–			
PR-P3	.13	.20*	–		
NR-P3	–.04	–.08	.67⁎⁎⁎	–	
ΔP3	.22⁎⁎	.35⁎⁎⁎	.58⁎⁎⁎	–.21*	–

Note. *p < .05

⁎⁎ *p* < .01

⁎⁎⁎ *p* < .001

Hierarchical regression analyses were conducted to examine the predictive effects of ERP indices (Table 3, Table 4). Covariates (anxiety and depression) showed no significant association with pornography use (R² = 0.037, *p* = .076). Adding PR-P3 or NR-P3 did not significantly improve model fit (ΔR² ≤ 0.019, *ps* > .10). In contrast, ΔP3 significantly improved prediction of pornography use (ΔR² = 0.046, *p* = .011) and emerged as a significant positive predictor (*β* = 0.214, *p* = .011).

**Table 3:** Hierarchical regression results predicting pornography use.

Predictor	Step 1 (Covariates)	Step 2a (NR-P3)	Step 2b (PR-P3)	Step 2c (ΔP3)
BDI	.135	.135	.136	.137
BAI	.080	.077	.083	.064
NR-P3		–.029		
PR-P3			.137	
ΔP3				.214*
Adjusted R^2^	.023	.017	.035	.062
ΔR^2^		.001	.019	.046
Δ*F*		0.12	2.70	6.73*
Overall *F*	2.63	1.78	2.67*	4.07⁎⁎

Note. Values are standardized regression coefficients (β). * p < .05

⁎⁎ p < .01.

**Table 4:** Hierarchical regression results predicting PPU symptoms.

Predictor	Step 1 (Covariates)	Step 2 (Pornography use)	Step 3a (NR-P3)	Step 3b (PR-P3)	Step 3c (ΔP3)
BDI	.103	.029	.029	.033	.039
BAI	.114	.070	.066	.075	.057
Pornography use		.549^⁎⁎⁎^	.548^⁎⁎⁎^	.531^⁎⁎⁎^	.497^⁎⁎⁎^
NR-P3			–.050		
PR-P3				.130	
ΔP3					.235^⁎⁎⁎^
Adjusted R^2^	.023	.313	.310	.325	.362
ΔR^2^		.291	.002	.017	.053
Δ*F*		58.38^⁎⁎⁎^	0.50	3.39	11.36^⁎⁎⁎^
Overall *F*	2.64	21.96^⁎⁎⁎^	16.54^⁎⁎⁎^	17.61^⁎⁎⁎^	20.58^⁎⁎⁎^

Note. Values are standardized regression coefficients (β). ^⁎⁎⁎^ p < .001

For PPU symptoms, pornography use was a strong predictor (ΔR² = 0.291, *p* < .001). Neither PR-P3 nor NR-P3 contributed additional explanatory power (ΔR² ≤ 0.017, *ps* ≥ .068). However, ΔP3 significantly improved model fit beyond covariates and pornography use (ΔR² = 0.053, *p* < .001), remaining a significant predictor of PPU severity (*β* = 0.235, *p* < .001).

### ROC curve analyses

**Fig. 3:**
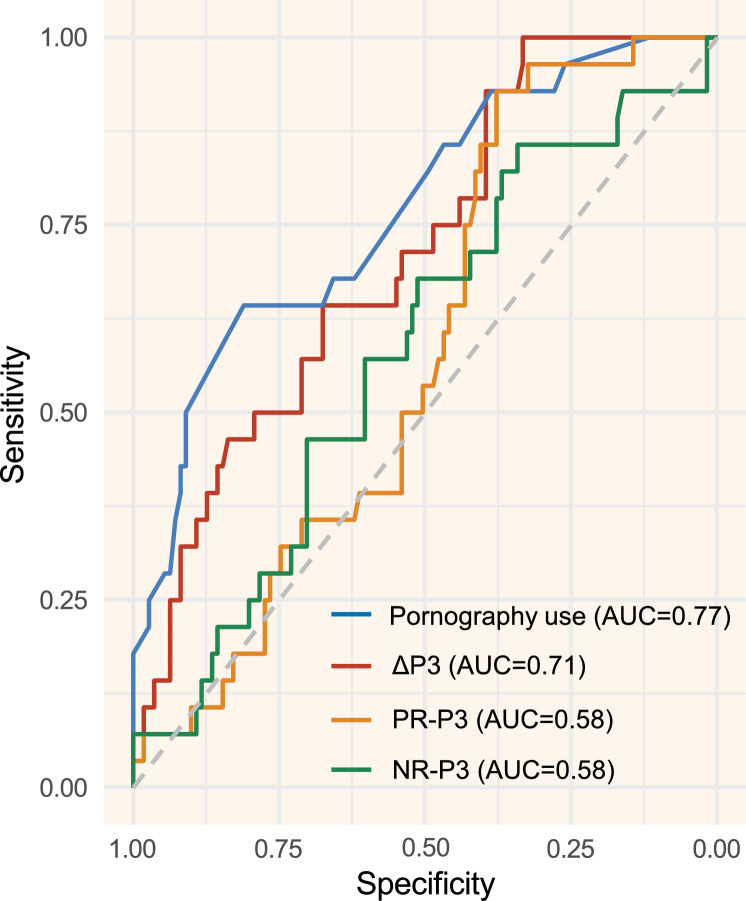
The receiver operating characteristic (ROC) curves for ΔP3, PR-P3, NR-P3, and self-reported pornography use in predicting PPU risk. The AUC values for each measure are as follows: ΔP3 (0.71), PR-P3 (0.58), NR-P3 (0.58), and pornography use (0.77).

ROC analyses indicated that ΔP3 showed acceptable discrimination for classifying high- versus low-risk PPU individuals (AUC = 0.71, 95% bootstrap CI [0.61, 0.81]; Fig. 3 and Supplementary Fig. 1). Cross-validated performance was highly consistent (mean CV AUC = 0.710), indicating stable out-of-sample generalization across repeated stratified folds. Examination of multiple cut-off thresholds revealed the expected sensitivity–specificity trade-off. ΔP3 showed relatively stable negative predictive value across thresholds, although positive predictive value remained modest (Table 5).

**Table 5:** Sensitivity, specificity, positive predictive value, and negative predictive value for the ΔP3 and pornography use measures in predicting PPU risk.

Predictor	Area Under the Curve	Cutoff	Sensitivity	Specificity	Positive Predictive Value	Negative Predictive Value
**ΔP3**	0.71	+2.0 SD	0.07	1.00	1.00	0.81
		+1.5 SD	0.14	0.96	0.44	0.82
		+1.0 SD	0.36	0.89	0.45	0.85
		+0.5 SD	0.50	0.72	0.31	0.85
**Pornography use**	0.77	+2.0 SD	0.21	0.97	0.67	0.83
		+1.5 SD	0.29	0.94	0.53	0.84
		+1.0 SD	0.39	0.92	0.55	0.86
		+0.5 SD	0.64	0.81	0.46	0.90

For comparison, pornography use demonstrated slightly higher but statistically comparable classification performance (AUC = 0.77, 95% bootstrap CI [0.67, 0.87]; mean CV AUC = 0.769). PR-P3 and NR-P3 showed substantially weaker discrimination (AUCs ≈ 0.58). Pairwise comparisons confirmed that ΔP3 significantly outperformed PR-P3 and NR-P3 (*ps* < .025), whereas no significant difference was observed between ΔP3 and pornography use (*p* = .398). These differences remained significant after Holm correction for multiple comparisons (both adjusted *p* = .042). Precision–recall analysis, which is sensitive to class imbalance, confirmed the same ordering of effects (pornography use > ΔP3 > NR-P3 ≈ PR-P3), indicating that model performance was not inflated by the unequal class distribution.

## Discussion

This study examined reward-related neural processing in PPU by analyzing P3 responses to pornographic and natural rewards, as well as their difference score (ΔP3). Consistent with reward imbalance models, ΔP3 was associated with both pornography use and PPU symptom severity and demonstrated discriminatory ability between high- and low-risk individuals. In contrast, PR-P3 and NR-P3 alone showed weak and non-significant associations with PPU outcomes. These findings suggest that relative, rather than absolute, neural responses to reward cues may be more informative for characterizing individual differences in PPU-related processes.

Broadly, these results are consistent with addiction models emphasizing altered reward processing, including increased sensitivity to addiction-related cues and reduced responsiveness to non-addictive rewards (Berridge & Robinson, 2016; Goldstein & Volkow, 2011; Koob & Volkow, 2016). Within this framework, compulsive sexual behavior has been conceptualized as involving dysregulated reward processing, although its classification remains under ongoing debate in current diagnostic systems (World Health Organization, 2020). The present findings align with the proposed dual-process accounts of addiction-related behavior (Dunning et al., 2011; Goldstein et al., 2008; Sescousse et al., 2013; Versace et al., 2012) and extend these models to PPU, supporting the relevance of reward imbalance perspectives in understanding compulsive sexual behavior (Brand et al., 2025; Klein et al., 2022; Stark et al., 2018).

Consistent with incentive sensitization theory (Robinson & Berridge, 1993), individuals at high risk for PPU showed a trend toward increased P3 responses to pornographic cues (PR-P3) compared to low-risk individuals. The P3 component reflects attentional allocation and the processing of motivational salience (Polich, 2007; Hajcak & Foti, 2020). Accordingly, a larger PR-P3 may indicate greater attentional engagement with pornographic stimuli in individuals with elevated PPU risk. This pattern is consistent with previous ERP and fMRI studies reporting enhanced cue reactivity in PPU (Draps et al., 2024; Gola et al., 2017; Voon et al., 2014; Wang et al., 2021). However, PR-P3 alone showed weak associations with PPU symptoms and limited classification performance, suggesting that cue reactivity in isolation may not fully capture individual differences in PPU. This is consistent with evidence from substance use disorders indicating that cue-reactivity measures alone are often insufficient predictors of clinical severity (Martins et al., 2022; Versace et al., 2012).

Although group differences in NR-P3 did not reach statistical significance, individuals at higher PPU risk showed a numerical reduction in responses to natural rewards. This pattern is broadly consistent with reward deficiency and allostatic models (Blum et al., 2012; Koob & Le Moal, 2008), although the effect appears subtle in the present sample. NR-P3 alone did not significantly predict PPU outcomes, suggesting limited utility as a standalone index. Instead, its relevance may be better understood in conjunction with responses to pornographic rewards, consistent with accounts emphasizing relative rather than absolute reward processing in addictive behaviors (Field et al., 2020; Monterosso et al., 2012; Vlaev et al., 2011).

The main finding of the present study is that ΔP3 (PR-P3 minus NR-P3) showed stronger and more consistent associations with PPU outcomes than either component alone. This pattern is broadly consistent with findings in substance use disorders, where indices of reward imbalance have been shown to outperform single-domain measures of cue reactivity or reward sensitivity (Martins et al., 2022). From a behavioral economic and neuroeconomic perspective, decision-making is shaped by relative valuation between competing rewards (Field et al., 2020; Monterosso et al., 2012; Vlaev et al., 2011). Addiction has been conceptualized as a state in which the relative value of addictive rewards may outweigh that of alternative natural rewards. In PPU, repeated exposure to highly salient sexual cues may bias reward valuation processes toward pornographic stimuli relative to alternative rewards, potentially contributing to motivational imbalance.

Crucially, the present findings should not be interpreted as evidence that ΔP3 constitutes a superior diagnostic instrument relative to self-report measures. Instead, ΔP3 reflects neural differentiation between responses to pornographic and natural rewards, capturing variation in relative reward processing that is not directly observable in behavioral frequency reports. Importantly, ΔP3 remained significantly associated with PPU symptoms even after controlling for pornography use and affective symptoms, indicating that it explains additional variance beyond self-reported behavior. From a measurement perspective, ΔP3 is best conceptualized as an index of relative reward processing at the level of value comparison between reward categories, whereas self-report measures primarily capture overt behavioral output and are additionally influenced by reporting-related factors such as recall limitations and response bias. These factors may contribute to partial divergence between neural and behavioral indices.

Consistent with this distinction, hierarchical regression analyses demonstrated that ΔP3 provided incremental explanatory power beyond self-reported pornography use. In ROC analyses, ΔP3 and pornography use showed comparable classification performance, suggesting that they carry similar amounts of discriminative information, although likely reflecting different underlying constructs. Importantly, ΔP3 showed robust discrimination across bootstrap and cross-validation procedures and significantly outperformed its constituent components (PR-P3 and NR-P3), supporting the added value of modeling relative rather than absolute reward responses.

The present findings have important theoretical implications. Overall, the results are consistent with models of PPU emphasizing altered reward processing and, more specifically, relative differences in reward responsiveness between pornographic and natural rewards, in line with evidence from substance-related and behavioral addictions (Martins et al., 2022; Sescousse et al., 2013). This supports dimensional accounts of compulsive sexual behavior within broader frameworks of addictive-like processes (Antons & Brand, 2021; Brand et al., 2025). Clinically, ΔP3 is not intended as a replacement for self-report measures, but as a complementary neural index that may provide additional information about reward-related processing. This may be particularly relevant in contexts where self-report is limited by recall bias or response tendencies. Although the present study is cross-sectional and does not permit causal inference, ΔP3 may serve as a potential mechanistic marker for future longitudinal and intervention research targeting reward-related processes. Interventions aimed at reward rebalancing, such as mindfulness-based approaches (Garland, 2024), natural reward training (Olsen, 2011), and cognitive remediation strategies (Anderson & Verdejo-Garcia, 2023), may benefit from incorporating neural indices of relative reward processing in future work.

Several limitations should be noted. First, the sample was restricted to young heterosexual male students, which may limit the generalizability of the findings. Given evidence of gender and sexual orientation differences in PPU (Bőthe et al., 2021, 2024; Grubbs et al., 2019), caution is warranted when extending the present results to other populations. Second, the cross-sectional design precludes causal interpretations. Although ΔP3 is discussed as reflecting individual differences in reward-related processing, the present data cannot determine whether neural reward imbalance precedes the development of PPU or emerges as a consequence of repeated exposure. Longitudinal research is needed to clarify temporal dynamics. Third, the study focused on a single ERP component (P3) measured at parietal sites, which provides limited insight into the broader neural circuitry of reward processing. Future studies combining ERP with fMRI or source localization approaches may help further elucidate the distributed neural systems underlying reward-related differences in PPU. Fourth, the operationalization of natural rewards was restricted to pleasant visual stimuli. Future work should incorporate a broader range of reward domains (e.g., monetary, social, or achievement-related rewards) to enhance ecological validity and better capture domain-general reward processing.

In conclusion, the present study provides evidence that differential neural responses to pornographic versus natural rewards are associated with individual differences in PPU and reflect relative reward processing between competing motivational systems. These findings are consistent with reward imbalance models of addiction and suggest that comparative neural indices may offer complementary information beyond single-cue reactivity measures. More broadly, the results contribute to understanding the neurocognitive mechanisms underlying PPU and support the relevance of reward-related processes in compulsive sexual behavior.

## Funding

This work was supported by grants from the 10.13039/501100013139Ministry of Education of Humanities and Social Science project (23YJC190021), and the Organized Research Project of Chengdu Medical College (CYYZZ25-22).

## Declaration of competing interest

The authors declare no conflict of interest.

## Appendix Supplementary materials

Image, application 1
