# Supplementary material for: Differential P3 reactivity to pornographic versus natural rewards as a neural marker of reward imbalance in problematic pornography use in young heterosexual men
Source: Int J Clin Health Psychol. 2026 Jul 24;26(2):100710. doi: 10.1016/j.ijchp.2026.100710 (PMC13444289; doi:10.1016/j.ijchp.2026.100710)
Supplement: Supplementary file 1 [file mmc1.docx]

**Differential P3 Reactivity to Pornographic versus Natural Rewards as a Neural Marker of Reward Imbalance in Problematic Pornography Use in Young Heterosexual Men**

**Supplementary Materials**

**Screening Instruments**

**Beck Depression Inventory–II (BDI-II; Beck et al., 1996)**

The BDI-II is a 21-item self-report questionnaire designed to assess the severity of depressive symptoms over the past week. Each item is rated on a 4-point scale ranging from 0 to 3, with higher total scores indicating more severe depressive symptoms. Total scores from 0–13 indicate minimal depression, 14–19 mild, 20–28 moderate, and ≥29 severe depression. In the present study, participants scoring 20 or above were excluded.

**Beck Anxiety Inventory (BAI; Beck et al., 1988)**

The BAI consists of 21 items assessing common symptoms of anxiety experienced during the past week. Items are rated on a scale from 0 (not at all) to 3 (severely), with total scores ranging from 0 to 63. Scores of 0–7 indicate minimal anxiety, 8–15 mild, 16–25 moderate, and 26–63 severe anxiety. Participants with scores of 16 or above were excluded.

**Fagerström Test for Nicotine Dependence (FTND; Fagerström, 1978)**

The FTND is a 6-item scale assessing the degree of physical dependence on nicotine. Items include cigarette consumption, time to first cigarette after waking, and difficulty refraining from smoking. Total scores range from 0 to 10, with scores ≥6 indicating high nicotine dependence. Participants exceeding this cutoff were excluded.

**Alcohol Use Disorders Identification Test (AUDIT; Babor et al., 2001)**

The AUDIT is a 10-item screening tool developed by the World Health Organization to assess hazardous and harmful alcohol use. Items are rated on a 5-point scale (0–4). Total scores range from 0 to 40; scores ≥8 suggest risky alcohol use, and participants above this threshold were excluded from the study.

**Internet Gaming Disorder Scale (IGDS; Pontes & Griffiths, 2015)**

The IGDS is a 9-item measure based on the DSM-5 criteria for Internet Gaming Disorder. Each item is rated on a 5-point Likert scale from 1 (never) to 5 (very often), with higher scores indicating greater severity of gaming-related problems. A total score ≥36 suggests a high likelihood of gaming disorder. Participants above this threshold were excluded.

**Problem Gambling Severity Index (PGSI; Ferris & Wynne, 2001)**

The PGSI is a 9-item instrument assessing gambling behaviors and their consequences over the past 12 months. Items are rated from 0 (never) to 3 (almost always). Total scores of 8 or above indicate problem gambling. Participants scoring in this range were excluded.

**Stimulus properties**

Thirty male undergraduate students who did not participate in the main experiment rated all images in a pilot study on 9-point scales for valence (1 = very unpleasant, 9 = very pleasant) and arousal (1 = not arousing, 9 = highly arousing). The three stimulus categories differed significantly from each other in both valence (pornographic: *M* = 5.77; pleasant: *M* = 7.04; neutral: *M* = 4.75) and arousal (pornographic: *M* = 6.58; pleasant: *M* = 5.50; neutral: *M* = 3.06).

**Table S**1. Intercorrelations Between Pornography Use, Psychiatric Symptoms, and Addictive Behaviors

| **Variable** | **1** | **2** | **3** | **4** | **5** | **6** | **7** | **8** |
| --- | --- | --- | --- | --- | --- | --- | --- | --- |
| 1. Pornography use | — |  |  |  |  |  |  |  |
| 2. PPU symptoms | .566^***^ | — |  |  |  |  |  |  |
| 3. BAI | .159 | .175^*^ | — |  |  |  |  |  |
| 4. BDI | .182^*^ | .170^*^ | .588^***^ | — |  |  |  |  |
| 5. FTND | .074 | .101 | .329 | .119 | — |  |  |  |
| 6. AUDIT | -.061 | .014 | .214 | .026 | .569^**^ | — |  |  |
| 7. IGDS | .109 | .101 | .069 | .197^*^ | .072 | .062 | — |  |
| 8. PGSI | .006 | .058 | .041 | .139 | -.242 | .048 | .153 | — |

*Note.* Pornography use = weekly viewing frequency × average duration per session; PPU symptoms = score on the Problematic Pornography Consumption Scale (PPCS); BAI = Beck Anxiety Inventory; BDI = Beck Depression Inventory; FTND = Fagerström Test for Nicotine Dependence; AUDIT = Alcohol Use Disorders Identification Test; IGD = Internet Gaming Disorder Scale; CPGI = Problem Gambling Severity Index.

^*^*p* < .05, ^**^*p* < .01, ^***^*p* < .001.

**Figure S1. Receiver operating characteristic (ROC) curve for ΔP3 in classifying high- versus low-risk PPU**

The red line represents the ROC curve for ΔP3, and the shaded area indicates the 95% bootstrap confidence interval. The diagonal line denotes chance-level classification (AUC = 0.50). The area under the curve (AUC) was 0.711 (95% bootstrap CI [0.614, 0.807]).
